# Subjective impact of osteoarthritis flare-ups on patients' quality of life

**DOI:** 10.1186/1477-7525-3-14

**Published:** 2005-03-16

**Authors:** Giuseppina Majani, Anna Giardini, Aurelio Scotti

**Affiliations:** 1 Psychology Unit, Fondazione S. Maugeri, Clinica del Lavoro e della Riabilitazione, IRCCS, Istituto Scientifico di Montescano (PV), Italy; 2 Scientific Department, Italfarmaco SpA, Milan, Italy

## Abstract

**Background:**

Clinical trials on osteoarthritis (OA) flare-ups treatment usually focus only on objective measures of health status, albeit recent literature suggestions on the importance of patients' subjectivity. Aim of the study was to evaluate the effects of OA and of its different types of medical treatment(s) on Health Related Quality of Life (HRQoL) in terms of both subjective satisfaction and functional status.

**Methods:**

An observational study on prospective data collected from the Evaluation of Quality of life in OA (EQuO) clinical trial (April 1999-November 2000) was conducted; outpatients from 70 participating centers (Orthopedy or Rheumatology Departments in Italy) with a diagnosis of OA of the hip or knee were consecutively enrolled. Patients were observed at OA flare-ups (baseline) and at follow up 4 weeks after treatment. Patients' objective and subjective HRQoL were assessed by means of the SF-36 and the Satisfaction Profile (SAT-P, which focuses on subjective satisfaction); Present Pain at baseline and Pain Relief at follow up were also evaluated.

**Results:**

Among the 1323 patients, 1138 (86%) were prescribed one drug/treatment of osteoarthritis, 169 (13%) 2 drugs/treatments, and 16 (1%) 3 drugs/treatments; most of treatments involved the prescription of NSAIDs; non-coxib, COX2 selective NSAIDs were prescribed in about 50% of patients. Follow-up visits were performed after 29.0 days on average (± 7.69 SD). For all SF-36 domains, all SAT-P items and factors, the differences between baseline and follow up scores resulted statistically significant (p < 0.001), enlighting an improvement both in health status and in subjective HRQoL.

**Conclusion:**

Besides the classic health status measures, the assessment of patients' subjective satisfaction provides important clues on treatments efficacy of OA within the patient-centered medicine model. In clinical practice this could lead to a better doctor-patient communication and to higher levels of treatment adherence.

## Background

The impact of osteoarthritis (OA) on patient's functional levels is well known [1-3]. Pain and physical limitations constitute difficulties patients have to deal with [4,5] and require long term pharmacological treatment and physical therapies.

Usually OA affects elderly people, and is one of the main causes of physical disability. In OA patients, Health Related Quality of Life (HRQoL) and activities of daily living are negatively affected. Significant work disability, reduced ability to deal with household duties and sleep disorders are reported in patients with symptoms of OA flare-ups, together with dysfunctions in the areas of ambulation, body-care and movement (in terms of perceived health status), and emotional behaviour (in terms of perceived psychological functioning) [1,2,4-8].

As a chronic condition, the impact of OA has been studied mainly focusing on its consequences on health status. Similarly, treatment efficacy is assessed within the context of health status and/or symptomatology in many clinical trials [6,7,9-12]. However, health status and symptomatology can be considered only two components of HRQoL [13] and little is known about the impact of OA and its treatments on patient's subjective perspective, in spite of increasing attention on this topic [14-19].

In literature, HRQoL refers to patients' appraisals of their current levels of functioning and satisfaction, compared to what they perceive to be ideal [20]. HRQoL assessment allows a subject to express his or her ability to perform daily activities across many domains which include physical, social and cognitive functioning, role activities and emotional wellbeing. Besides, "...how a subject feels about the performace of each of those activities may be assessed separately by measuring satisfaction for each domain." [21]. The subjective implications of HRQoL, within the context of patient centred medicine, have been already stressed by suggestions from recent reliable scientific literature [15,17-24].

The aim of the present study is to evaluate the effects of OA and of its different types of medical treatment(s) on HRQoL in terms of both subjective satisfaction and functional status.

## Methods

### Patient population and procedure

Data from collaborating, educated outpatients aged 50–80 years with a diagnosis of OA of the hip or knee according to the criteria of the American College of Rheumatology [25] were collected in this observational, prospective study.

Outpatients (n = 1340) were consecutively enrolled in 70 Italian participating centers (Orthopedy or Rheumatology Departments, listed in Appendix A [see additional file]) from April 1999 to November 2000. 147 patients withdrawn OA treatment before follow-up visit.

All patients signed an informed consent in which the purposes of the study (HRQoL assessment and treatment efficacy, as primary and secondary outcomes respectively) were clearly stated. Approval for this research was obtained by the ethics committee, patients did not receive any remuneration for their participation.

Patients with concomitant osteoarticular disorders, impairment of motor function not due to OA of the hip or knee, concomitant systemic disease(s) affecting HRQoL or requiring NSAIDs/steroids use on a regular basis were not included into the study, in order to avoid biases in the results due to treatments other than OA treatments.

Patients were observed at OA flare-ups, when attending for a visit (baseline) and at follow up 4 weeks after treatment. According to the observational design of this trial, no "study treatments" were assigned to patients, but any drug(s)/medical treatment(s) considered by the physician as adequate to the patient's clinical condition was freely prescribed; therefore patients were not previously randomized to treatment.

During both visits, patients were administered the following: the Visual Analogue Scale (VAS) [26] on Present Pain (baseline) or on Pain Relief (follow up); the Medical Outcomes Study Short-Form 36 Health Status Survey (SF-36) [27] in its validated Italian version [28,29] and the Satisfaction Profile (SAT-P) [30].

Moreover, at follow up, the global assessments of efficacy and tolerability of the medical treatment(s) prescribed for OA flare ups (expressed by the patient and by the physician according to a 4 point semi-quantitative rating scale: excellent – good – moderate – poor) were collected. Side effects to this/these treatment(s), if any, were registered as well.

The assessment procedure was standardized for all the participating centres. During the visit patients were invited to compile alone all the questionnaires and rating scales, only if required patients were assisted by a trained health professional.

Self-reporting bias in HRQoL improvements was kept under control by the assessment procedure and by the adoption of valid and reliable questionnaires.

### Measures

#### Visual Analogue Scale

The VAS is perhaps the most widely used instrument for the measurement of pain intensity. The classic version of the VAS was administered: 10 centimeter line, horizontal. "It is a simple, robust, sensitive, and reproducible instrument that enables a patient to express the severity of his pain in such a way that it can be given a numerical value." [26] Its psychometric properties and its utility in clinical trials have been confirmed [2,8,31,32]. VAS on Present Pain ranged from "no pain" to "the worst pain possible"; VAS on Pain Relief ranged from "no pain relief" to "the maximum pain relief". Scores ranged from 0 to 100.

#### SF-36

The SF-36 is a well known self-administered and generic health status measure which encompasses 8 domains related to daily life activities: physical functioning, role limitations due to physical problems, role limitations due to emotional problems, vitality, bodily pain, social functioning, mental health and general health perception [33-35]. Each domain scores from 0 (lowest level of functioning) to 100 (highest level of functioning). The instrument has been extensively validated within the Medical Outcome Study [33] and in other settings [34].

#### Satisfaction Profile

The SAT-P is a self-administered, generic questionnaire which provides a satisfaction profile in daily life and can be considered as an indicator of subjective QoL. Satisfaction can be defined as the cognitive product of the comparison between ideal life and reality, and can therefore be quantitatively measured. The subject is asked to evaluate his/her satisfaction about 32 life aspects with reference to the last month (on 32 10 cm horizontal VAS) independently of his/her objective health status (for example: "How satisfied have you been in the last month with your Resistance to physical fatigue?"; "How satisfied...with your Mood?"; "How satisfied...with your Emotional stability?"). It provides 32 individual scores and 5 factor scores, all ranging from 0 (lowest level of satisfaction) to 100 (highest level of satisfaction). Together with its ability to detect patient's subjective satisfaction, the SAT-P addresses some aspects of daily life which are not included in SF-36 items (i.e. sleep, sexual life, quality of couple relationship, eating, self-confidence, resistance to stress, etc.). Its psychometric properties and clinical utility have been confirmed [30,36,37].

### Statistical analyses

Sociodemographic data and clinical values were analysed by means of descriptive statistics. Since the incidence of withdrawals resulted low, analyses were performed on complete cases and no solutions for handling missing data was adopted.

Baseline and follow-up of SF-36 and SAT-P item and factor scores were compared by means of Analusis of Covariance (ANCOVA). Moreover, ANCOVAs were adopted in order to evaluate the impact of clinical variables on SF-36 and SAT-P factor delta scores (calculated subtracting the follow-up scores from baseline scores). The variables included into the models were: age, gender, body weight, OA localization (hip, knee, or both), VAS Present Pain, presence of concomitant disease(s), type of treatment (COX2 selective NSAIDs vs. other treatments). Results were summarized using mean ± SE for continuous variables and frequency (absolute and percent) for categorical variables. All p values are two-tailed and p < .05 was considered statistically significant. All computations were carried-out by resorting to SAS 8.0 procedures.

## Results

Patients demographic and clinical characteristics (OA localization (hip/knee/both), VAS Present Pain, type of medical treatment(s) of OA flare-ups, concomitant diseases and treatments are shown in Table 1. Patients' baseline VAS Present Pain resulted consistent with a clinical condition of moderate to severe rheumatic disease.

**Table 1:** Patients' characteristics

Gender (F/M)	795/528
Age (years, mean ± SD)	64.4 ± 10.3
Marital status:	
Single, n (%)	72 (5.4)
Married, n (%)	922 (69.7)
Widowed, n (%)	220 (16.6)
Separated/divorced, n (%)	13 (1.0)
Missing, n(%)	96 (7.3)
Educational level:	
Primary school, n (%)	548 (41.4)
Junior high school, n (%)	325 (24.6)
Senior high school, n (%)	280 (21.2)
Degree/Master/PhD, n (%)	103 (7.7)
Missing, n(%)	67 (5.1)
Employment status:	
Employed, n (%)	434 (32.8)
Retired, n (%)	550 (41.6)
Housewife, n (%)	288 (21.8)
Missing, n (%)	51 (3.8)
Body weight (kg, mean ± SD)	73.4 ± 11.0
OA localization:	
Knee, n (%)	658 (49.7)
Hip, n (%)	463 (35.1)
Knee + hip, n (%)	202 (15.2)
VAS Present Pain (mm, mean ± SD)	67.7 ± 17.0
Concomitant diseases, n (%)	632 (47.8)
Concomitant treatments, n (%)	444 (33.6)

The most frequent concomitant diseases were: hypertension (19.1%), metabolic and nutritional disorders (9.2%), muscoloskeletal, connective tissue and bone disorders (8.2%) and gastrointestinal system disorders (4.3%). The most frequently prescribed concomitant treatments were: cardiologic drugs (9.7%) and antihypertensive (9.4%), antidiabetic drugs (8.4%), antithrombotic agents (4.5%), antiacids (6.7%), sedatives (4.8%). 1138 patients (86%) were prescribed one drug/treatment of OA, 169 patients (13%) 2 drugs/treatments, and 16 patients (1%) received 3 drugs/treatments. Most of treatments involved the prescription of NSAIDs; non-coxib, COX2 selective NSAIDs (nimesulide betadex and nimesulide, the only two COX2 selective NSAIDs available in Italy at the time of this study) were prescribed in about 50% of patients (Table 2).

**Table 2:** Treatments prescribed for osteoarthritis flare-ups

	n	% patients
COX2 NON-SELECTIVE NSAIDs		
Arylacetic acid derivatives (diclofenac, indomethacin, sulindac, etc.)	221	16.7
Arylpropionic acid derivatives (ibuprofen, naproxen, ketoprofen, etc.)	165	12.5
Oxycams (piroxicam, tenoxicam, etc.)	181	13.7
Others (nabumetone, glucosamine, diacerein, etc.)	107	8.1
COX2 SELECTIVE NSAIDs		
Nimesulide betadex (or nimesulide)	689	52.1
OTHER DRUGS/TREATMENTS		
Various, systemic (ASA, paracetamol, corticosteroids, centrally acting myorelaxants)	46	3.5
Various, topical (transcutaneous or intraarticular)	44	3.3
Physical treatment (mobilization, iontophoresis, etc.)	60	4.5

Follow-up visits were performed after 29.0 days on average (± 7.69 SD). Only a small number of patients (17; 1.2%) did not attend follow-up visit.

### HRQoL assessment: SF-36

For all SF-36 domains, the difference between baseline and follow up scores resulted statistically significant (p < 0.001) (Table 3).

**Table 3:** SF 36 scores (Mean ± SE). Baseline vs Follow-up scores. At the ANCOVAs: p < 0.001 for all domains

SF-36 domains	Baseline	Follow up	p
Physical Functioning	47.9 ± 0.7	59.3 ± 0.7	<.001
Role Physical	27.8 ± 1.0	48.0 ± 1.1	<.001
Bodily Pain	31.7 ± 0.4	50.5 ± 0.5	<.001
General Health	45.8 ± 0.5	50.0 ± 0.5	<.001
Vitality	46.5 ± 0.5	53.2 ± 0.5	<.001
Social Functioning	44.1 ± 0.6	65.4 ± 0.6	<.001
Role Emotional	46.9 ± 1.2	65.7 ± 1.1	<.001
Mental Health	59.2 ± 0.5	65.4 ± 0.5	<.001

Baseline Present Pain was associated with almost all the SF-36 domains (Table 4). The presence of concomitant disease(s) resulted in a statistically significant association with 4 domains: Role Physical, Bodily Pain, General Health, Social Functioning. The type of OA treatment was associated with Physical Functioning and Bodily Pain. OA localization and age was associated with only one domain: Physical Functioning and Role Physical respectively. Gender and body weight did not correlate with any SF-36 domain.

**Table 4:** Detected statistical significances on SF-36 delta scores. The p values resulted from the ANCOVAs are indicated.

SF-36 domains	Covariates						
	Age	Gender	Body weight	OA localization	Present Pain	Concomitant diseases	Treatment
Physical Functioning				0.021	0.0001		0.020
Role Physical	0.022					0.007	
Bodily Pain					0.0001	0.0001	0.006
General Health					0.0001	0.003	
Vitality					0.0001		
Social Functioning					0.0001	0.003	
Role Emotional					0.007		
Mental Health					0.0001		

### HRQoL assessment: SAT-P factors

All the differences between baseline and follow up SAT-P factor scores were statistically significant (p < 0.001) (Table 5).

**Table 5:** SAT-P factor scores (M ± SE). Baseline vs Follow-up scores. At the ANCOVAs: p < 0.001 for all Factors.

SAT-P Factors	Baseline	Follow up	p
Psychological functioning	59.3 ± 0.6	65.5 ± 0.5	p <.001
Physical functioning	41.3 ± 0.5	51.9 ± 0.5	p <.001
Work	53.3 ± 0.7	57.8 ± 0.7	p <.001
Sleep/Eating/Leisure	55.4 ± 0.5	60.9 ± 0.5	p <.001
Social functioning	66.0 ± 0.6	70.8 ± 0.5	p <.001

Baseline pain was significantly associated with all SAT-P factors (Table 6). The presence of concomitant disease(s) was in a statistically significant association with 3 out of 5 factors: Psychological functioning, Sleep-Eating-Leisure, Social functioning. OA treatment was associated with the factor Sleep-Eating-Leisure.

**Table 6:** Detected statistical significances on SAT-P factors. The p values resulted from the ANCOVAs are indicated

SAT-P Factors	Covariates						
	Age	Gender	Body weight	OA localization	Present Pain	Concomitant diseases	Treatment
Psychological functioning					0.0001	0.022	
Physical functioning					0.0001		
Work					0.007		
Sleep/Eating/Leisure					0.0001	0.007	0.026
Social functioning					0.0001	0.018	

### HRQoL assessment: SAT-P items

**Figure 1:**
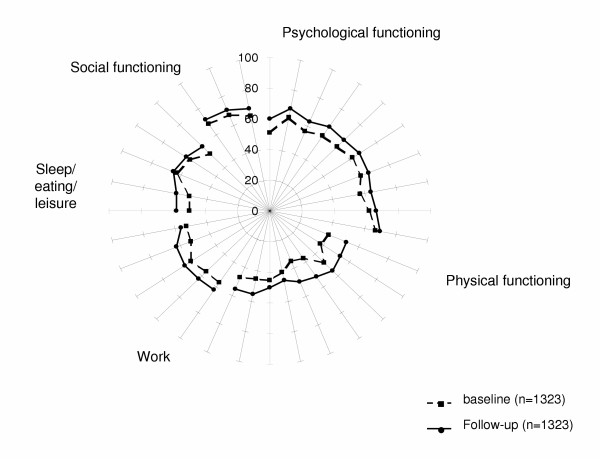
SAT-P items: mean scores at baseline and at follow-up. For all the differences (ANCOVAs) p < 0.001.

Figure 1 shows the graphic representation of baseline and follow up SAT-P item scores. All the differences were statistically significant (p < 0.001).

### Clinical outcome of OA treatment: Efficacy and Tolerability

At follow-up, mean VAS Pain Relief was 61.1 mm (± 24.3 SD).

In 65% of cases treatment efficacy was evaluated as good or excellent by patients themselves, in 67% of cases it was evaluated as good or excellent by physicians. In 81% of cases treatment tolerability was evaluated as good or excellent by patients themselves, in 84% of cases it was evaluated as good or excellent by physicians. It was evaluated as poor in 7% and 6% of cases respectively (Table 7).

**Table 7:** OA treatments' evaluations (efficacy and tolerability)

	Poor	Moderate	Good	Excellent	Missing data
	n (%)	n (%)	n (%)	n (%)	n (%)
Efficacy – patients	163 (12.3)	288 (21.8)	634 (47.9)	227 (17.2)	11 (0.8)
Efficacy – physicians	129 (9.8)	292 (22.1)	634 (47.9)	257 (19.4)	11 (0.8)
Tolerability – patients	93 (7.1)	147 (11.1)	712 (53.8)	359 (27.1)	12 (0.9)
Tolerability – physicians	79 (6.0)	122 (9.2)	703 (53.1)	407 (30.8)	12 (0.9)

11.1% of patients reported side effects to medical treatment of OA; most of these reactions involved the gastrointestinal system. Poor tolerability led to treatment withdrawal in 6.2% of patients.

## Discussion

Our study represents, to our knowledge, the largest observational prospective clinical trial carried out in OA patients' subjective HRQoL. The sample size and the very small number of drop-outs could be considered the strenghts of the study.

A limit of the study could be considered the adoption of the SAT-P which is a new questionnaire, validated on the Italian population [30], but not previously used in clinical trials or in OA patients. Nevertheless, its psychometric properties have been previously confirmed, and moreover it is the only Italian questionnaire specifically aimed at assessing subjective satisfaction in daily life, independently of the presence of a disease. Its user friendly structure and its easily comprehensible graphical representation could be considered substantial methodological facilities both in research and in clinical practice.

Finally, the coherence between the data provided by the two HRQoL instruments could confirm that health status and subjective satisfaction partially overlap, and allows us to study the same phenomenon from two different points of view: the objective and the subjective. This could therefore be considered the added value of the study.

Considering the whole sample, SF-36 results confirm what previous studies have already enlightened in clinical trials: the SF-36 is, according to Kosinski et al. [35], a suitable instrument for assessing health status in OA, and medical treatment improves functionality levels in daily life aspects.

The same conclusions could be drawn for the SAT-P: on the whole sample a general improvement of satisfaction levels can be observed in all the 32 items considered. In other words, pharmacological treatment has a significant positive impact on patients' both objective functioning and subjective well-being [16].

Thanks to the sinergic utility of the two instruments it has been possible to enlight results otherwise left unperceived and whose positive value on patients' life is unquestionable.

Further investigations are needed in order to better clarify the relationships between perceived pain and pain relief and patients' HRQoL. Mastery, self-efficacy and coping abilities could be significant mediators between these two constructs [38,39].

## Conclusion

From both an objective and a subjective point of view, OA flare-ups' treatment has proved to have positive effects on HRQoL. The sinergic use of a health status measure (SF-36) and of a tool addressing subjective satisfaction (SAT-P) allows to wider the focus on patients' life.

This methodological approach could help clinicians and researchers in transferring into practice the ICF model issues [40], with special attention on Activity and Participation and on Environmental Factors.

## List of abbreviations

ANCOVA Analysis of Covariance

HRQoL Health Related Quality of Life

NSAID Non Steroid Anti Inflammatory Drugs

OA Osteoarthritis

SAT -P Satisfaction Profile

SF-36 Medical Outcome Study Short-Form 36 Health Status Survey

VAS Visual Analogue Scale

## Competing interests

The author AS is employee of the company that partially funded the study.

## Authors' contributions

GM: responsible for the design of the study, contributed to the statistical evaluation, contributed to the writing of the paper.

AG: responsible for the statistical evaluation, contributed to the writing of the paper.

AS: contributed to the design of the study and data collection, contributed to the statistical evaluation, contributed to revise the manuscript.

## Supplementary Material

Additional File 1 Appendix A – Participating Centers Click here for file
