# Supplementary material for: Subjective impact of osteoarthritis flare-ups on patients' quality of life
Source: Health Qual Life Outcomes. 2005 Mar 16;3:14. doi: 10.1186/1477-7525-3-14 (PMC555754; doi:10.1186/1477-7525-3-14)
Supplement: Additional File 1 — Appendix A – Participating Centers [file 1477-7525-3-14-S1.doc]

# Appendix A - Partecipating Centers

- Dr. Salvatore ALFIERI

Ospedale di MARATEA (PZ)

- Dr. Antonio AMATULLI

Ospedale di NOCI (BA)

- Dr. PierPaolo ARMANDO

Ospedale “Gravina”

CALTAGIRONE (CT)

- Dr. Antonello ASUNI

Ospedale Sirai – CARBONIA (CA)

- Dr. Pierpaolo BACCHIDDU

Ospedale Marino

CAGLIARI (CA)

- Dr. Domenico BORRI

Ospedale Civile

di SULMONA (AQ)

- Dr. Vito BORROMETI

ROMA

- Dr. Eugenio BOUX

Ospedale Civile di CHIVASSO (TO)

Dr. Rolf BUSCH

Ospedale SS.Annunziata di

SAVIGLIANO (CN)

- Dr. Francesco CANTISANI

Ospedale S.Giuseppe

EMPOLI (FI)

- Dr. Giulio CASTELLANI

Ospedale Torrette

di ANCONA (AN)

- Dr. Luca CASTELLI

Ospedale Civile Orlandi

BUSSOLENGO (VR)

- Dr. Fabio CATALANO

ASL Roma G di TVOLI (RM)

- Dr. Angelo CATANIA

Ospedale Civile “Abele Ajello”

di MAZARA DEL VALLO (TP)

- Dr. Claudio CITTI

Ospedale Civile di

CITTA’ DI CASTELLO (PG)

- Dr. Aldo DE AMICIS

Ospedale Fatebenefratelli

MILANO

- Dr. Alessandro DE FABRITIS

Ospedale S.Giuseppe

MARINO (RM)

- Dr. Giovanni D’ELIA

Ospedale Civile di Gaeta

ASL di FORMIA (LT)

- Dr.ssa Marina DE LISA

Distretto Sanitario 91-ASL SA 1

di NOCERA INFERIORE (SA)

- Dr. Corrado DENARO

Ospedale di AVOLA (SR)

- Dr. Vito DE TULLIO

Osp. “SS.Antonio e Biagio-.Arrigo”

ALESSANDRIA (AL)

- Dr. Efisio ESPA

Ospedale di OZIERI (SS)

- Dr. Alberto FEDERICI

Ospedale di RECCO (GE)

- Dr. Fabrizio FRANCO

Az.Osped.”Villa Sofia”

PALERMO (PA)

- Dr. Vincenzo GALLENTI

Ospedale Civile di

VITTORIA (RG)

- Dr. Paolo GHIGGIO

Ospedale di IVREA (TO)

- Dr. Fabio GIANCECCHI

Ospedale di MACERATA

- Dr. Sergio GIGLIOTTI

ASL NA1 – NAPOLI

- Dr. Raffaele GIORDANO

Ospedale di TREVIGLIO (BG)

- Dr. Guido GIUSSANI

Ospedale di GARBAGNATE (MI)

- Dr. Giuseppe GRAZIANO

Presidio Ospedaliero di

TERMINI IMERESE (PA)

- Prof. Pasquale GRECO

Ospedale di CASERTA

- Dr. Pierangelo GUZZENTI

Ospedale di TARQUINIA (VT)

- Dr. Calogero INGRAO

Ospedale S.Spirito

CASALE MONFERRATO (AL)

- Dr. Walter LEONARDI

Ospedale S.Vincenzo

TAORMINA (ME)

- Dr. Pierfrancesco LEUCCI

Poliambulatorio di MAGLIE (LE)

- Dr. Mario LONGO

Ospedale di SASSUOLO (MO)

- Dr. Giovanni MARGIACCHI

Ospedale di FIGLINE V.NO

(FI)

- Dr. Alvise MARTON

Ospedale di DOLO (VE)

- Dr. Giancarlo MELIS

Ospedale Marino

ALGHERO (SS)

- Dr.ssa M.Teresa MERCURI

Poliambulatorio U.S.L. 13 –AP

ACOLI PICENO (AP)

- Dr. Filippo MORSIANI

Ospedale di SCANDIANO (RE)

- Dr. Pierfrancesco MURENA

Ospedale Sant’Antonio

SAN DANIELE DEL FRIULI (UD)

- Dr. Aldo MUSCOLINO

Ospedale Civile di

PARTINICO (PA)

- Dr. Paolo PALOMBI

Ospedale di TIVOLI

- Dr. Ottavio PANFILI

Ospedale di MIRANO (VE)

- Dr. Salvatore PIU

Ospedale di MURAVERA (CA)

- Dr. Arcangelo POPOLIZIO

Fondazione Don Gnocchi

POZZOLATICO (FI)

- Dr. Elio RAGAZZI

Ospedale di MIRANDOLA (MO)

- Dr. Otello REGENI

Ospedale Civile di

PALMANOVA (UD)

- Dr. Vincenzo RICCI

Ospedale di OSIMO (AN)

- Dr. Alessandro ROSSI

Dist. U.S.L. di NAVACCHIO (PI)

- Dr. Giacomo ROTILIO

Ospedale di PALESTRINA (RM)

- Dr. Fausto SALAFFI

Ospedale Civile

Di JESI (AN)

- Dr. Giuseppe SANTE’

ASL 1 – NAPOLI

- Dr. Rosario SARDINA

Ospedale S.Antonio Abate

ERICE CASA SANTA (TP)

- Dr. Giacinto SCIGHILONE

Ospedale di MONTEROTONDO (RM)

- Dr. Alessandro SOLDINI

Ospedale di VOGHERA (PV)

- Dr. Oliviero SORAGNI

Ospedale di BORGO MAGGIORE

Repubblica di San Marino

- Dr. Giulio SORRENTINO

Ospedale Civile di

S.GAVINO (CA)

- Dr. Roberto STORACI

Ospedale di LUINO (VA)

- Dr. Marco TABARRONI

Presidio Ospedaliero

BENTIVOGLIO (BO)

- Dr.ssa Tatiana TOMMASIN

Ospedale S.Luca

TRECENTA (RO)

- Dr. Luigi TORELLI

Ospedale di Niguarda

MILANO (MI)

- Dr. Claudio VANNI

Terme di Casciana S.p.A.

CASCIANA TERME (PI)

- Prof. Claudio VELLUTI

Ospedale di MARINO (CA)

- Dr. Francesco VISENTINI

Ospedale di MONSELICE (PD)

- Dr. Paolo VIZZIELLO

Ospedale ASL n.4 di MATERA (MT)

- Dr. Joshi WAMAN

STRADELLA (PV)

- Dr. R. ZINI

Az.Osped.”San Salvatore”

PESARO (PS)
